# Spatially-Resolved Thermometry of Filamentary Nanoscale Hot Spots in TiO_2_ Resistive Random Access Memories to Address Device Variability

**DOI:** 10.1021/acsaelm.3c00782

**Published:** 2023-09-05

**Authors:** Timm Swoboda, Xing Gao, Carlos M. M. Rosário, Fei Hui, Kaichen Zhu, Yue Yuan, Sanchit Deshmukh, Çaǧıl Köroǧlu, Eric Pop, Mario Lanza, Hans Hilgenkamp, Miguel Muñoz Rojo

**Affiliations:** † Department of Thermal and Fluid Engineering, Faculty of Engineering Technology, University of Twente, Enschede 7500 AE, The Netherlands; ‡ Faculty of Science and Technology and MESA+ Institute for Nanotechnology, University of Twente, Enschede 7500 AE, The Netherlands; § School of Materials Science and Engineering, Zhengzhou University, Zhengzhou 450001, China; ∥ MIND, Department of Electronic and Biomedical Engineering, Universitat de Barcelona, Barcelona 08007, Spain; ⊥ Materials Science and Engineering Program, Physical Science and Engineering Division, King Abdullah University of Science and Technology (KAUST), Thuwal 23955-6900, Saudi Arabia; # Department of Electrical Engineering, Stanford University, Stanford, California 94305, United States; % Department of Materials Science and Engineering, Stanford University, Stanford, California 94305, United States; & Precourt Institute for Energy, Stanford University, Stanford, California 94305, United States; @ 2D Foundry, Instituto de Ciencia de Materiales de Madrid (ICMM), CSIC, Madrid 28049, Spain

**Keywords:** resistive random access memory, scanning thermal microscopy, device variability, conductive filaments, heat dissipation in electronics

## Abstract

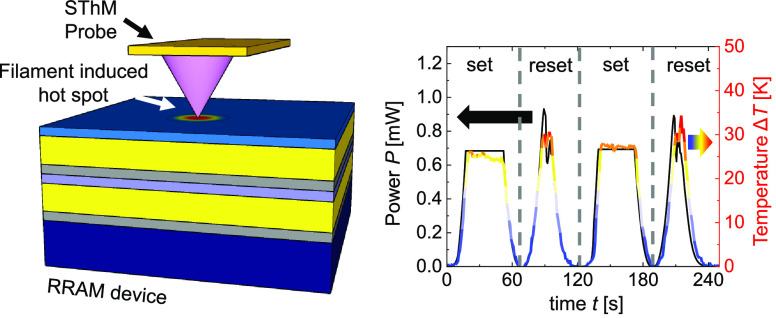

Resistive random access memories (RRAM), based on the formation and rupture of conductive nanoscale filaments, have attracted increased attention for application in neuromorphic and in-memory computing. However, this technology is, in part, limited by its variability, which originates from the stochastic formation and extreme heating of its nanoscale filaments. In this study, we used scanning thermal microscopy (SThM) to assess the effect of filament-induced heat spreading on the surface of metal oxide RRAMs with different device designs. We evaluate the variability of TiO_2_ RRAM devices with area sizes of 2 × 2 and 5 × 5 μm^2^. Electrical characterization shows that the variability indicated by the standard deviation of the forming voltage is ∼2 times larger for 5 × 5 μm^2^ devices than for the 2 × 2 μm^2^ ones. Further knowledge on the reason for this variability is gained through the SThM thermal maps. These maps show that for 2 × 2 μm^2^ devices the formation of one filament, i.e., hot spot at the device surface, happens reliably at the same location, while the filament location varies for the 5 × 5 μm^2^ devices. The thermal information, combined with the electrical, interfacial, and geometric characteristics of the device, provides additional insights into the operation and variability of RRAMs. This work suggests thermal engineering and characterization routes to optimize the efficiency and reliability of these devices.

## Introduction

Resistive switching devices are considered promising for nonvolatile memory and neuromorphic computing.^1−3^ A type of such devices is resistive random access memory (RRAM), which tends to feature low power consumption, high speed, and simple device configuration.^4^ RRAM devices typically consist of an oxide insulator sandwiched in a two-terminal metal–insulator–metal layered structure.^5^ The principle of operation of an RRAM device is based on the formation (set) and breaking (reset) of a conductive filament in the oxide layer.^6,7^ In the recent past, resistive switching has been investigated in a wide variety of metal oxides, like HfO_2_,^8−10^ Ta_2_O_5_,^11,12^ or TiO_2_.^13−15^ Some of the major challenges associated with these devices are related to a lack of reliability in device operation and storage mechanisms that results in high variability of their electrical performance.^16,17^ Understanding the underlying fundamental operation, like filament size,^18^ position,^19^ current density,^20^ and heating,^21^ is therefore essential for the evaluation, design, and optimization of RRAMs. Different studies have estimated that the diameter of conductive filaments could be below 10 nm.^22,23^ Through these confined conductive regions flow large currents that can lead to high power densities >10^13^ W/cm^3^.^9^ Deshmukh et al.^9^ determined that these high power densities can cause extremely high temperature rise, over 1000 K in HfO_2_-based RRAM filaments. These elevated temperatures not only reduce the endurance and performance of devices themselves but also threaten the operation of the electronics in the vicinity because of potential thermal crosstalk. Within this context, thermal management is becoming essential in memory circuits, like those for neuromorphic computing, where controlling temperature variation is needed for efficient and stable data processing.^24^ Therefore, further observations and analyses of filament-induced hot spots in RRAMs are relevant for achieving optimum, reliable, and efficient performance.

The need to improve the reliability of RRAM devices has been a topic of ample discussion.^25−27^ Park et al.^26^ characterized the existence of multiple conductive paths in Ta_2_O_5–*x*_/TaO_2–*x*_ RRAM devices by means of transmission electron microscopy (TEM). However, on the basis of these measurements, no conclusions can be drawn on the heat distribution associated with these filament-based devices during operation. Baeumer et al.^27^ observed the change of the position of the active conductive filament as a consequence of *I*–*V* cycling in SrTiO_3_ RRAM devices using photoelectron emission microscopy (PEEM). The filament is localized through the analysis of the photoemission threshold difference across the device area. PEEM is limited by its depth of field of a few nanometers and by the lack of information toward the current or heat distribution in steady state. Because of the depth limitation, the PEEM studies must also be performed on special device structures, for example, by using photoelectron-transparent graphene electrodes.^27^

Scanning thermal microscopy (SThM)^28−34^ is a scanning probe microscopy (SPM)-based technique that offers new possibilities to explore the operation of electronic devices. It uses a special temperature sensitive probe with high precision (<1 K) that enables the characterization of thermal phenomena with nanoscale spatial resolution. SThM has been applied to study the energy dissipation of different devices, like memories,^9,35,36^ and phase change materials (PCM).^30,37^ Importantly, the combination of high thermal and spatial resolution makes SThM an ideal tool for analyzing heating in filament-based RRAM devices.^34,38^ As an example, Datye et al.^35^ employed SThM for surface mapping of the hot spots due to conductive bridges formed in MoTe_2_ memory devices. Recently, Deshmukh et al.^9^ imaged the spatial extent and temperature of the filament operation in HfO_*x*_-based RRAMs, assessing the effect of heat spreading on memory operation. Similarly, Nandi et al.^36^ investigated the temperature distribution in NbO_*x*_-based RRAM devices. Additional studies on the fundamental thermal behavior of filamentary memories are essential for gaining further insight toward how the switching mechanisms are influenced by geometry, materials, and contacts. This will enable new thermal engineering routes for more efficient and reliable RRAMs.

In this work, we used SThM to characterize the localized filamentary heating in TiO_2_-based cross-point RRAM devices and correlate these observations with device performance and reliability. While previous reports using SThM analysis on memories^9,35,36^ focus on the fundamental assessment of filament-induced heat spreading, the evaluation of the memory switching variability by combining electrical and thermal data has not previously been carried out. The analysis of the thermal maps obtained by SThM is capable of providing further insights into the cause of device switching variability. We chose TiO_2_ as the switching material for the RRAM, given its widespread use for these devices and because it is reliable and is easy to grow, which makes it a good candidate for the characterization of different areas.^39,40^ We observed significant differences in the thermal behavior of devices with two different cross-point areas, i.e., 2 × 2 and 5 × 5 μm^2^, in terms of their *I*–*V* switching variability and the stability of the conductive filament. SThM provides valuable information to evaluate sources of variation and to suggest routes for optimizing device performance and reducing variability.

## Experimental Results

### Fabrication of the RRAM Device Structure

**Figure 1:**
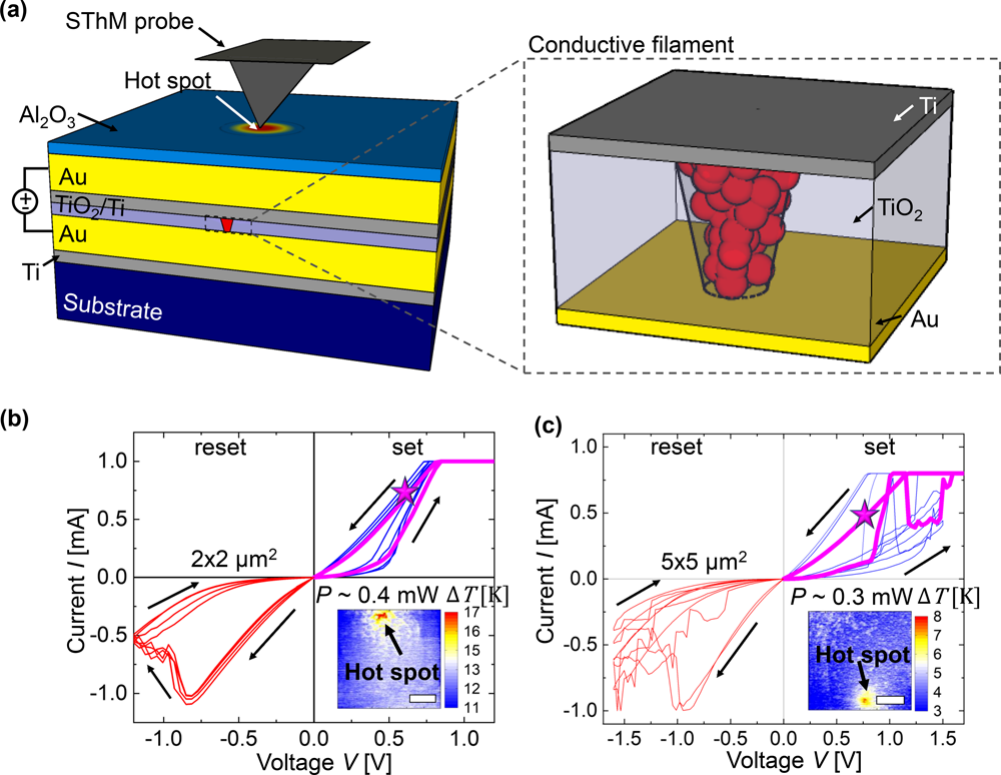
(a) Cartoon diagram of the device and measurement setup, showing the SThM probe on top of the RRAM. The zoom-in schematic shows the conductive filament formed in the active TiO_2_/Ti layer upon application of an electrical bias *V*. (b, c) Measured *I–V* characteristics of the devices for four cycles. The inset figures at the bottom right show the SThM temperature map of the same device at the bias point corresponding to the star symbol. The thermal maps reveal the hot spot generated by the filament for a device area of (b) 2 × 2 μm^2^ (scale bar 350 nm) and (c) 5 × 5 μm^2^ (scale bar 1 μm).

Figure 1 shows a cross-sectional schematic of the investigated RRAM structure based on a thin TiO_2_/Ti layer (from bottom to top) sandwiched between two Au electrodes. To fabricate this RRAM, we first deposited a thin 10 nm Ti layer for adhesion on top of a Si/SiO_2_ (300 nm) substrate. Then, we used e-beam evaporation to deposit the bottom Au electrodes with a thickness of 30 nm. The switching material consists of a TiO_2_/Ti bilayer where each layer has a thickness of 10 nm, deposited by e-beam evaporation (Ti) and atomic layer deposition (TiO_2_). A 30 nm thick top Au electrode was evaporated with e-beam. Finally, we covered the whole structure with a 10 nm thick Al_2_O_3_ capping layer grown by atomic layer deposition (ALD) to electrically insulate the sample. The top view of the device consists of a cross-point structure with contact pad sizes of 100 × 100 μm^2^. For the purpose of this study, we fabricated devices with two different cross-point areas: 2 × 2 and 5 × 5 μm^2^ (see Supporting Information Section S1).

### Characterization of the Current–Voltage Characteristics

The filament formation process in metal oxide RRAMs is achieved when applying a forming voltage (up to 3 V) to the device, which is associated with the creation of a conductive path that results from the connection of oxygen vacancies.^1^ Forming is accompanied by a sharp decrease of the device resistance from native oxide resistance to a low resistive state (LRS). The filament can be (partially or completely) broken when a sufficiently high reverse bias (−1.5 V < *V* < −1 V) is applied to the device (reset process), causing an increased resistance also referred to as high resistive state (HRS). The device can switch to LRS on applying a voltage, lower than during forming (0.5 V < *V* < 1.5 V), showing the bipolar nature of our devices (set process). The electrical measurements were performed in a probe station connected to a Keithley 4200 A-SCS semiconductor parameter analyzer (SPA) applying voltage or current bias at room temperature. In pristine TiO_2_ RRAMs described above, we formed the filament by applying a positive voltage sweep with an initial current compliance of *I*_cc_ = 1 μA. After initial forming, we repeated these measurements (*I*_cc_ ≤ 1 mA) for ten cycles to ensure cyclability of the devices (see Supporting Information Section S2). Figure 1 show two examples of multiple *I–V* curves obtained at two devices with an area of (b) 2 × 2 μm^2^ and (c) 5 × 5 μm^2^. In both examples we observed the previously mentioned sharp increase and decrease of the electric current during the set and reset processes, respectively. After measuring more than 40 devices both electrically and thermally, we observed a higher cycle-to-cycle variability in the 5 × 5 μm^2^ devices compared to the 2 × 2 μm^2^ ones, as can be seen in Figure 1b,c. Additionally, we observed a higher intrinsic device-to-device variability in the 5 × 5 μm^2^ devices (see Supporting Information Section S3).

### Thermal Characterization with SThM

SThM measurements were performed on our RRAMs to simultaneously image the topography and heating features on the device surface, while electrical bias is applied to the device. To obtain thermal maps with SThM, we used an Asylum atomic force microscope (AFM) and an SThM thermoresistive probe (Pd on SiN from Bruker). These SThM probes can correlate temperature variations in the tip to changes in electrical resistance with Δ*R*_probe_ ∝ Δ*T*_probe_.^41^ The SThM probe is electrically connected to an external Wheatstone bridge consisting of two fixed resistances (1 kΩ each), a potentiometer (*R*_pot_), and the resistance of the probe (*R*_probe_). When the SThM is operated, a voltage bias is applied to the Wheatstone bridge to induce an electric current. The potential measured across the bridge (*V*_SThM_) allows us to determine accurately the changes of the electrical resistance of the probe and, hence, temperature variations on the surface of the device. Note that the electric current through the probe causes Joule self-heating and an increase in probe temperature, which consequently results in an increase in its electrical resistance. For thermal sensing, this current must be as low as possible to keep the probe self-heating low compared to the temperature of the surface being scanned. During SThM scans, a constant electrical bias was applied between the two electrodes of the RRAM device. The conversion of SThM electrical probe signals into surface temperature to determine the heating of the RRAM devices is possible because of a process of calibration. For more details about the calibration, we would like to refer to ref (42) and Supporting Information Section S4.

As illustrated in Figure 1a, after the conductive filament is formed in the RRAM device, SThM can map the hot spot generated on the surface of the device due to Joule heating. In the insets of Figure 1b,c, we present two steady-state temperature maps with their corresponding *I*–*V* curves. The star symbol indicates the voltage bias applied to the device while performing the SThM scan. In both cases, we observed a hot spot with a surface temperature increase of Δ*T* = 17 and 8 K in the 2 × 2 and the 5 × 5 μm^2^ device, respectively, when powers of ∼400 μW and ∼300 μW were applied. We recall that this temperature was measured at the surface; therefore, it is not the internal temperature of the filaments, and one needs to consider that the heat generated by the filament in the metal oxide also spreads along the top and bottom electrodes. Therefore, both the temperature and the size of the heating spot at the surface differ from those of the buried filament, as shown by Deshmukh et al.^9^

### Steady-State Measurements

**Figure 2:**
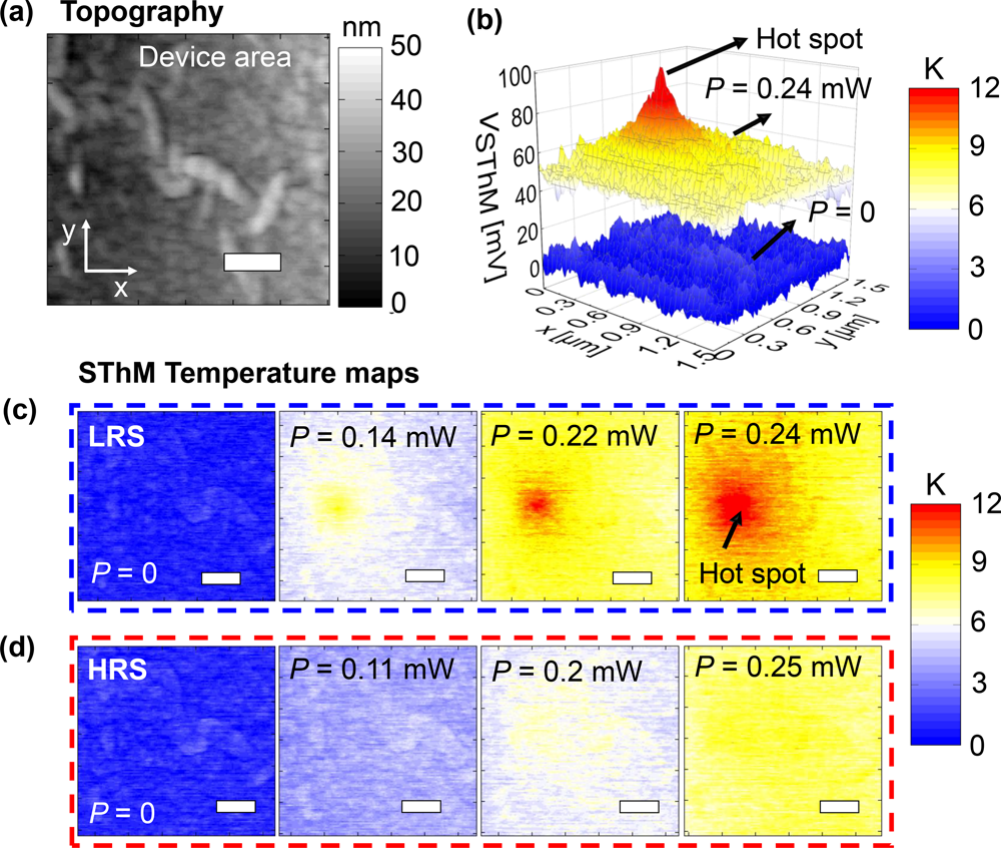
(a) Topographic image of a device with an area of 5 × 5 μm^2^ (scale bar 300 nm). (b) SThM thermal signal *V*_SThM_ (*z*-axis) map obtained for the device shown in (a) when applying a power of *P* = 0.24 mW and under no power applied (*P* = 0). The difference in SThM signal between heated and nonheated case is converted into a temperature change (Supporting Information Section S4), as represented by the color scheme. (c, d) SThM temperature maps with four different power levels after (c) setting the device at positive polarity (blue dashed rectangle) and after (d) resetting the device at negative polarity (red dashed rectangle) (scale bar = 300 nm).

Figure 2a shows the topography of a RRAM device scanned with an SThM probe. The topography of the devices can vary in their surface roughness. However, we observed the peak of the heating independently from the topographical artifacts (Supporting Information Section S5). Figure 2b shows a 3D representation of two thermal maps obtained for a 5 × 5 μm^2^ device at 0 mW power and at 0.24 mW in its LRS. During the first scan at the bottom, we scanned the cross-point area while no power is applied to the device, so the device is not heated. For the second map above, we applied a power of *P* = 0.24 mW to the device. In this case, a current of *I* ∼ 0.4 mA passes through the filament, which results in a localized hot spot on the surface. Considering a stable filament resistance, the magnitude of the resulting surface temperature mostly depends on the power and polarity of the electric current applied to the device.

Figure 2 shows multiple temperature maps obtained on the device presented in Figure 2b after the device was set (c) vs reset (d). In the LRS the hot spot is localized at the same location, and its temperature scales up as the power applied to the device increases (same LRS, i.e., no cycling between images). We observed that the maximum hot spot temperature increased from 9 K at 0.14 mW to 14 K at 0.24 mW. The power itself does not impact the shape of the hot spot, but it increases the temperature. After reset, we obtained temperature maps for similar powers as in the set case but with reversed polarity (see Figure 2d). Considering the higher resistance in the reset, we applied higher voltages than in the LRS. At a power of 0.11 mW, we barely see any visible localized heating. At higher power we observed mostly uniform heating on the device, with little localized heating that could eventually be related to a partial but not complete breaking of the filament during the reset process. As an example, in Figure 2d at 0.2 mW, it looks like the breaking of the filament is not fully complete, as minor heating is still visible at the initial position of the filament. At sufficiently high power we observed a partial set of the device in agreement with the *I*–*V* characteristics. In the 2 × 2 μm^2^ device of Figure 1b, we observed a softer breaking of the filament in accordance with the *I*–*V* cycling behavior of the device (see Supporting Information Section S5).

The results in Figure 2 present the temperatures of the RRAM devices at the surface. For the characterization of the filament temperature, we employed an electrothermal simulation in COMSOL Multiphysics (see Supporting Information Section S6). Thus, we estimated the relevant material and contact characteristics in order to fit the temperature profiles of the maps in Figure 2 and the corresponding potential measured in our devices. As a result, we calculated the maximum filament temperature rise to be between 172 and 245 K above the ambient temperature, depending on the power applied to the device ranging from 0.14 to 0.24 mW (see Supporting Information Section S7).

### In-Operando SThM Measurements at the Hot Spot

**Figure 3:**
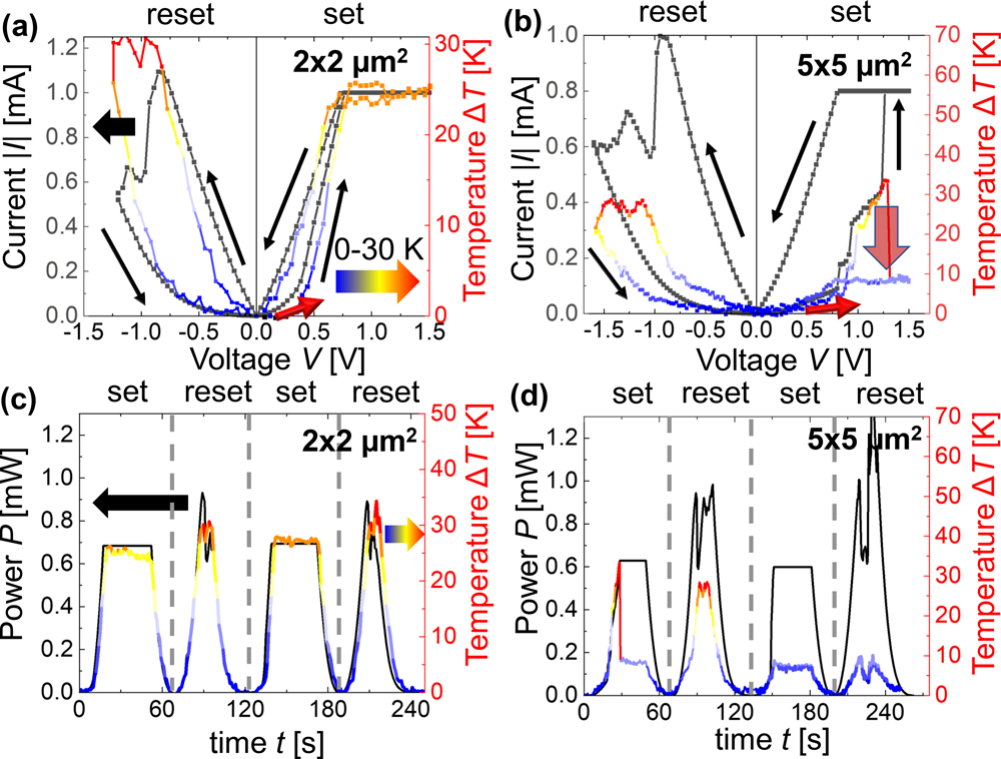
(a, b) Absolute electric current |*I|* (left axis) and temperature increase Δ*T* (right axis) for one cycle (set and reset) as a function of the sweeping voltage for a device area of (a) 2 × 2 μm^2^ and (b) 5 × 5 μm^2^. The red arrows indicate the beginning of the sweep. (c, d) Electrical power (left axis) and temperature increase (Δ*T*, right axis) for two full cycles of set and reset as a function of time for a device area of (c) 2 × 2 μm^2^ and (d) 5 × 5 μm^2^. The color scale of the temperature graphs ranges from 0 K (blue) to ∼30 K (red).

Next, we aimed to correlate the heating of the hot spot with the operando electrical *I*–*V* behavior of the devices during cycling. For that purpose, we kept the SThM probe static at the position of the hot spot, which we localized during the steady-state measurements, while running *I*–*V* sweeps on the device. The SThM software provides a logger option that records the operando SThM thermal signal as a function of time. This approach allows investigation of how the hot spot on the surface heats depending on the operando power applied. Figure 3 shows the *I*–*V* measurements for devices with an area of (a) 2 × 2 μm^2^ and (b) 5 × 5 μm^2^. The right axes of these figures show the surface temperature measured by the SThM, which increases as the power of the devices rises. The temperature evolution in Figure 3a,b for the 2 × 2 and 5 × 5 μm^2^ devices, respectively, follows the same trend as the *I*–*V* curve for low voltages. However, we observed a drop in the temperature in the 5 × 5 μm^2^ device illustrated by the partially transparent arrow during set, which is not in line with the *I*–*V* curve obtained at higher voltages. Simultaneously the current sharply increases, which corresponds to the shift from HRS to LRS. With the aim to obtain more insights into this observation, we compared the SThM temperature measurements with the electrical power applied to the device.

Figure 3 shows the power and Δ*T* over different cycles of set and reset. Figure 3c shows that the heating measured by the SThM probe in the original position of the hot spot for the 2 × 2 μm^2^ device is consistent for different cycles of set and reset. This is indicative of having a reliable filament that forms and breaks in the same device location, which we observed reliably for >10 devices. However, Figure 3d shows that the heating around the original filament location for the 5 × 5 μm^2^ device varies during set and reset. This is indicative of position variation of the conductive filament, and it was observed in five devices. To further analyze this observation, we characterized how the steady-state measurements of the same devices as displayed in Figure 3 varied with cycling.

### Steady-State Characterization after Cycling

**Figure 4:**
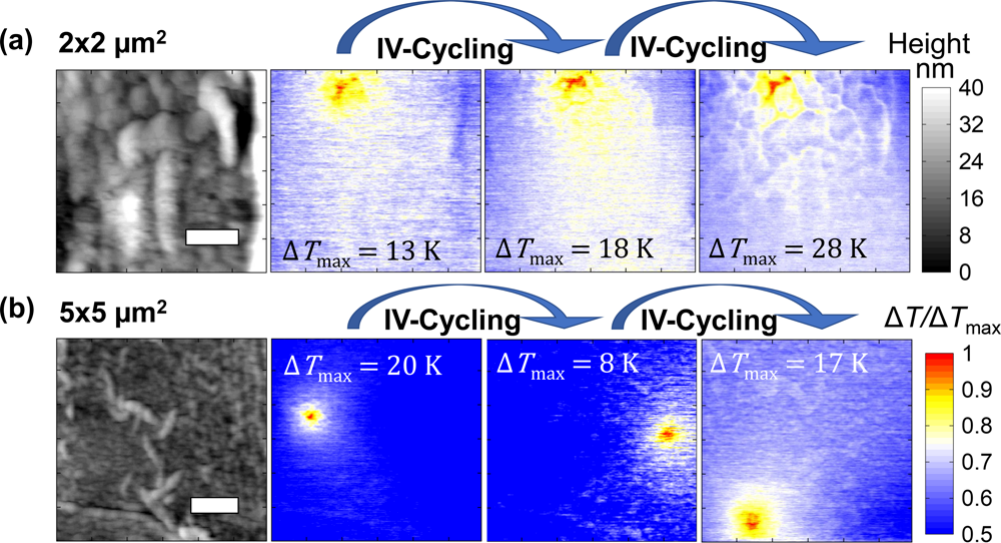
Topography (left) and surface temperature maps of the device (right) with area of (a) 2 × 2 μm^2^ and (b) 5 × 5 μm^2^ during steady-state measurements. The gray scale represents the topographical changes and the colored scale the temperature changes in these maps. Power *P* applied to the devices during the scans in (a) are 0.21, 0.4, and 0.57 mW and in (b) these are 0.39, 0.15, and 0.37 mW with a maximum temperature as labeled on each plot. The device power is the product of the voltage across and the current flowing through the device; differences in the resistance of the filament therefore affect the electrical power values.

In order to determine whether the location of the hot spot varies between set and reset cycles, we performed steady-state thermal maps of the same devices after *I*–*V* sweeps. To verify a constant tip position during in-operando measurements, we used the same tip offset in the steady-state measurements before and after cycling. Figure 4 shows temperature maps together with the topographic image of the device for the two areas under study, i.e., (a) 2 × 2 μm^2^ and (b) 5 × 5 μm^2^. Each device was set and reset multiple times, and the steady-state thermal maps were obtained in the LRS between cycles.

The maps of the 2 × 2 μm^2^ device reveal that the position of the hot spot and thus of the filament remained unchanged after cycling (Figure 4a). However, we observed a shift of the hot spot location in the maps of the 5 × 5 μm^2^ device with an average distance of 1.6 μm between each other (Figure 4b). These SThM observations enable evaluation of the cycle-to-cycle variation by measuring the shift of the filament position in RRAM devices, which cannot be done by electrical measurements.

## Discussion

With the thermal information provided here, we can observe the heat distribution on the surface of relevant RRAM devices during the set and reset processes. As an example, from Figure 4b we can see that the hot spot is moving, which relates to the formation of filaments in separate locations. The electrical information combined with the thermal SThM analysis displayed in this work for TiO_2_ RRAM devices provides information relevant to the parameters that affect their reliability. In this case, the electrical and thermal signatures of the device allow us to draw conclusions on how the geometry and electrical connections affect the operation. More specifically, for a better understanding of these results, we discuss the differences in heating between the 2 × 2 and 5 × 5 μm^2^ devices, considering (i) the area of the devices and (ii) the resistance of the top and bottom metal lines that connect the device with the electrode pads, which is equivalent to a series resistor.

First, we observed a shift of the hot spot location in five different devices with an area of 5 × 5 μm^2^. The distance between each hot spot (distance between temperature peak) varied between 1.5 and 3 μm with an average distance of 2.21 ± 0.87 μm, and there were up to three possible hot spot locations with reversible switching at similar power conditions (see Supporting Information Section S8). This average distance between hot spots is larger than the size of the 2 × 2 μm^2^ devices. Therefore, we suggest that the size of the device could be a limiting factor for the filament to relocate, being more favorable to form just one filament in smaller devices, <2 × 2 μm^2^ (as hypothesized before).^43^

Second, an additional perspective correlates with the different widths of the metal lines connecting the top and bottom electrode with the pads. The size of the metal lines in the 2 × 2 μm^2^ devices is smaller than in the 5 × 5 μm^2^ devices, which results in a higher series resistance and lower capacitance. We measured the line resistance for both devices by applying a current between the electrode pad and the end of the electrode line. The resistance of the electrode metal lines for the 2 × 2 and 5 × 5 μm^2^ devices were 308 and 123 Ω, respectively. However, the total series resistance is higher, as can be estimated from the measured *I*–*V*, following the work of Fantini et al.^44^ From this estimation we obtained a total series resistance of around 1000 and 300 Ω for 2 × 2 and 5 × 5 μm^2^ devices, respectively (see Supporting Information Section S9). The remaining resistance could originate from the conduction through the Ti layer and TiO_2_/Ti interface, where a partial oxidation of the Ti occurs due to the oxygen exchange reaction.^45,46^ Using an integrated series resistance has been shown to be an effective method to decrease transient current overshoot in RRAM devices.^47,48^ The higher series resistance and lower capacitance in the 2 × 2 μm^2^ devices makes them more robust against current overshoot during electroforming and set events, which could be responsible for the change of the active filament position observed in the larger 5 × 5 μm^2^ devices.

Finally, in this work, we also analyzed the potential impact of cycling at the interfaces of the device. For that purpose, we employed a high-angle annular dark field scanning transmission electron microscope (HAADF-STEM) to characterize the device structure before and after filament forming and cycling. We used an energy-dispersive X-ray spectroscopy (EDX) detector to analyze the elemental distribution of the relevant elements (i.e., Ti, Au, and O) in the metal/insulator/metal structure (see Supporting Information Section S10). We observed a continuous interface between layers in the active area of the pristine devices. However, we observed small gaps at the interface between TiO_2_ and the Au bottom electrode for the cycled devices regardless of cross-point area. Similar observations were noted by Carta et al.^49^ in Pt/TiO_2_/Pt devices. They observed a delamination between the TiO_2_ and the top electrode for cycled devices, which they claimed to be induced from the O_2_ gas generated during the filament forming process. The delamination may contribute to the cycle-to-cycle and device-to-device variabilities and further lead to performance degradation in the RRAM devices, though more evidence is required. Despite this effect, our *I*–*V* characteristics and SThM images show that the device keeps switching during cycling with similar thermal and electrical characteristics. Therefore, we can conclude that the set and reset stem from the formation and breaking of the filament rather than being dominated by delamination effects.

## Conclusion

In conclusion, we used SThM to obtain surface temperature maps of TiO_2_ memory devices with multiple sizes, operating under both steady-state and operando conditions, to evaluate their heating features. The thermal insights obtained for the device combined with its electrical characteristics allowed us to correlate the reliability of the devices with their design parameters. The results obtained reveal that the position shift of filaments is a significant cause of the electrical variability in RRAM devices. Additionally, these results are indicative for the existence of multiple possible filament positions in specific designs of RRAM devices and demonstrated that heat dissipation can vary locally as a function of cycling. Future studies should continue analyzing the impact of the area, line resistance, and interfacial structure during cycling in other RRAM devices to gain a better understanding of how it affects their performance. On one hand, the SThM measurement approach presented in this study can be conveniently expanded for the thermal characterization of other filament-based switching memristive devices (e.g., unipolar and diffusive memristors). On the other hand, a more fundamental analysis of the forming process and filament features could also be addressed in the future, requiring carefully designed electrothermal experiments (e.g., measurements on various devices with different switching mechanisms) combined with filamentary electrothermal model analysis. Overall, SThM proves itself as a powerful approach to gain further insights into RRAM operation. This provides new routes for thermal and electrical characterization and engineering of RRAM not only restricted to metal oxide-based resistive switching.

## Supporting Information Available

Section S1: schematic of device structure with fabrication specifications; Section S2: *I–V* curves of devices with up to 50 full cycles; Section S3: *I*–*V* curves of devices that display cycle variability depending on the device area; Section S4: schematic and results of the SThM calibration approach; Section S5: SThM steady-state temperature maps of a 2 × 2 μm^2^ device for different power and resistive states; Section S6: COMSOL model used for the characterization of the filament temperature; Section S7: FEM temperature profiles of a hot spot surface temperature in comparison to experimental results; Section S8: 3D SThM temperature maps of two devices of different area size as a function of cycling; Section S9: *I*–*V* graphs of the electrodes for the characterization of the line resistance; Section S10: STEM micrographs and EDX maps of a pristine and cycled RRAM device; Section S11: *I*–*V* and logger temperature evolution graphs for two devices with higher cycle number (PDF)
