# Supplementary material for: Spatially-Resolved Thermometry of Filamentary Nanoscale Hot Spots in TiO2 Resistive Random Access Memories to Address Device Variability
Source: ACS Appl Electron Mater. 2023 Sep 5;5(9):5025–31. doi: 10.1021/acsaelm.3c00782 (PMC10537448; doi:10.1021/acsaelm.3c00782)
Supplement: Supplementary file 1 — el3c00782_si_001.pdf [file el3c00782_si_001.pdf]

Supporting Information: Spatially-resolved thermometry of filamentary nanoscale hot spots in TiO_2 resistive random access memories to address device variability

Timm Swoboda[1], Xing Gao[2], Carlos M. M. Rosário[2], Fei Hui[3], Kaichen Zhu[4], Yue Yuan[5] Sanchit Deshmukh[6], Çağıl Köroğlu[6], Eric Pop[6,7,8], Mario Lanza[5], Hans Hilgenkamp[2] and Miguel Muñoz Rojo[1,9,]*

[1]Department of Thermal and Fluid Engineering, Faculty of Engineering Technology, University of Twente, Enschede, 7500AE, The Netherlands

[2]Faculty of Science and Technology and MESA+ Institute for Nanotechnology, University of Twente, Enschede, 7500AE, The Netherlands

[3]School of Materials Science and Engineering, Zhengzhou University, Zhengzhou, 450001, China

[4]MIND, Department of Electronic and Biomedical Engineering, Universitat de Barcelona, Barcelona, 08007, Spain

[5] Materials Science and Engineering Program, Physical Science and Engineering Division, King Abdullah University of Science and Technology (KAUST), Thuwal 23955-6900, Saudi Arabia

[6]Department of Electrical Engineering, Stanford University, Stanford, CA, 94305, USA

[7]Department of Materials Science and Engineering, Stanford University, Stanford, CA, 94305, USA

[8]Precourt Institute for Energy, Stanford University, Stanford, CA, 94305, USA

[9]2D Foundry, Instituto de Ciencia de Materiales de Madrid (ICMM), CSIC, Madrid, 28049, Spain

*corresponding author: m.m.rojo@csic.es

S1 Fabrication of the samples

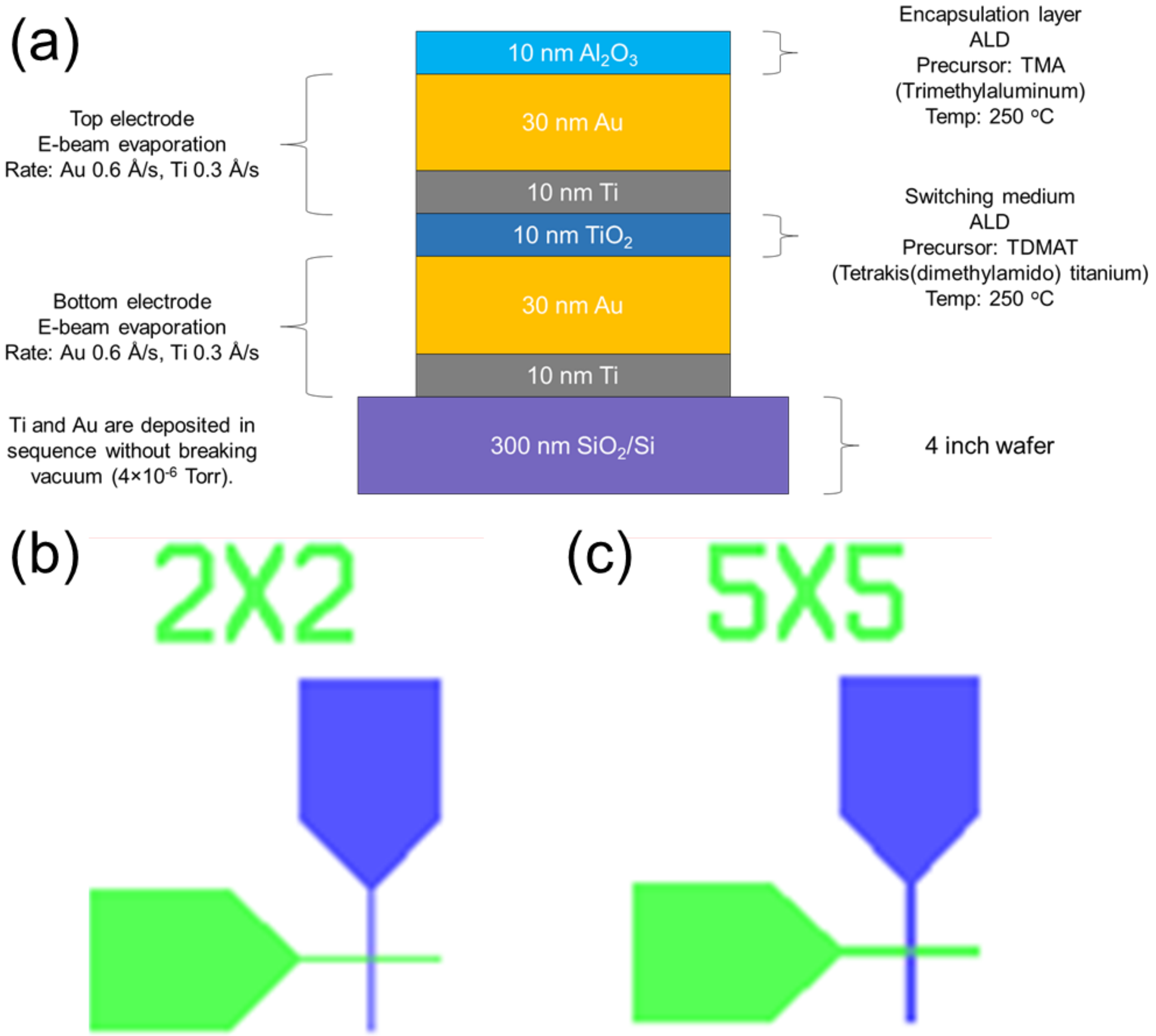

Figure S1. (a) Schematic of the fabrication procedure of the investigated samples. The thickness and fabrication method of each layer is indicated in the drawing. **(b,c)** Schematic of the cross-point structure for the (b) 2x2 μm^2 devices and (c) 5x5 μm^2 devices.

We fabricated our sample structure on top of a 300 nm SiO_2/Si substrate. We deposited 30 nm of Au as the bottom electrode on top of a 10 nm thick adhesion layer by means of e-beam evaporation. Then, we deposited the 10 nm TiO_2 active layer by means of atomic layer deposition (ALD) and 10 nm Ti through e-beam evaporation. After that, we deposited 30 nm Au top electrode by means of e-beam evaporation. For the purpose of electrical isolation, we capped the whole structure with a 10 nm thin Al_2O_3 capping layer. The details of the device layers are illustrated in Figure S1 (a). We arranged the devices in a cross-point structure with an active area size of (b) 2x2 and (c) 5x5 μm^2. The contact pad size is 100x100 μm^2 in both cases.

S2. Electrical characterization in RRAM

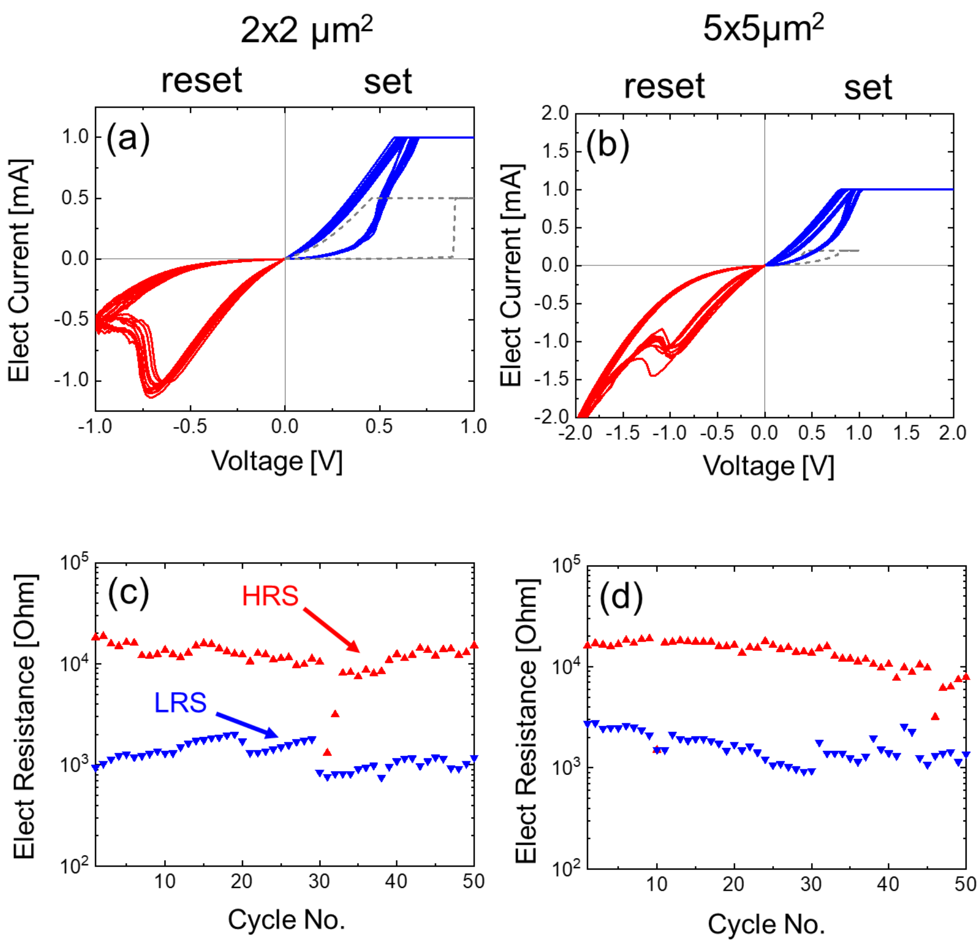

Figure S2. (a,b) Electric current as a function of the electrical voltage during the set (blue) and reset process (red) for 10 cycles in a (a) 2x2 μm^2 and in a (b) 5x5 μm^2 device. The grey dashed curves show the forming process. **(c,d)** Electrical resistance in the high resistive state (HRS) in red and the low resistive state (LRS) in blue of the RRAM devices vs number of cycles for a cross-point area size of (c) 2x2 μm^2 and (d) 5x5 μm^2.

For the electrical switching process of our resistive random access memory (RRAM) devices we performed electric voltage sweeps using a semiconductor parameter analyzer (SPA). Before forming the filament, we used an initial current compliance of I_{cc} = 1 μA, in order to decrease the severity of overshooting effects. However typically we required to use higher compliances in order to form the filament completely. The forming voltage $V_{forming}$ in between devices varied usually in the range of 0.5 to 2 V having a higher magnitude than the subsequent set voltage which is in line with observations in the literature.[1] After the forming process we reset the device by applying a reversed bias with a higher I_{cc} than in the last set or forming process. To ensure the cyclability of the devices we set and reset them for at least 10 cycles before we checked them with the scanning thermal microscope (SThM).

Figure S2 (a,b) show two examples of one 2x2 μm^2 and one 5x5 μm^2 devices representing ten full cycles. During the cycles we applied a constant current compliance during the set process to achieve consistent set conditions. Figure S2 (c,d) show the electrical resistance in the high resistive state (HRS) and the low resistive state (LRS) for two devices with a cross-point area of (c) 2x2 μm^2 and (d) 5x5 μm^2 during 50 full cycles. The HRS and LRS values were read at 0.07 V before and after the switching in the set process. The results support that our devices are capable of switching for more than 50 cycles.

S3. Device-to-device (D2D) and cycle-to-cycle (C2C) variability

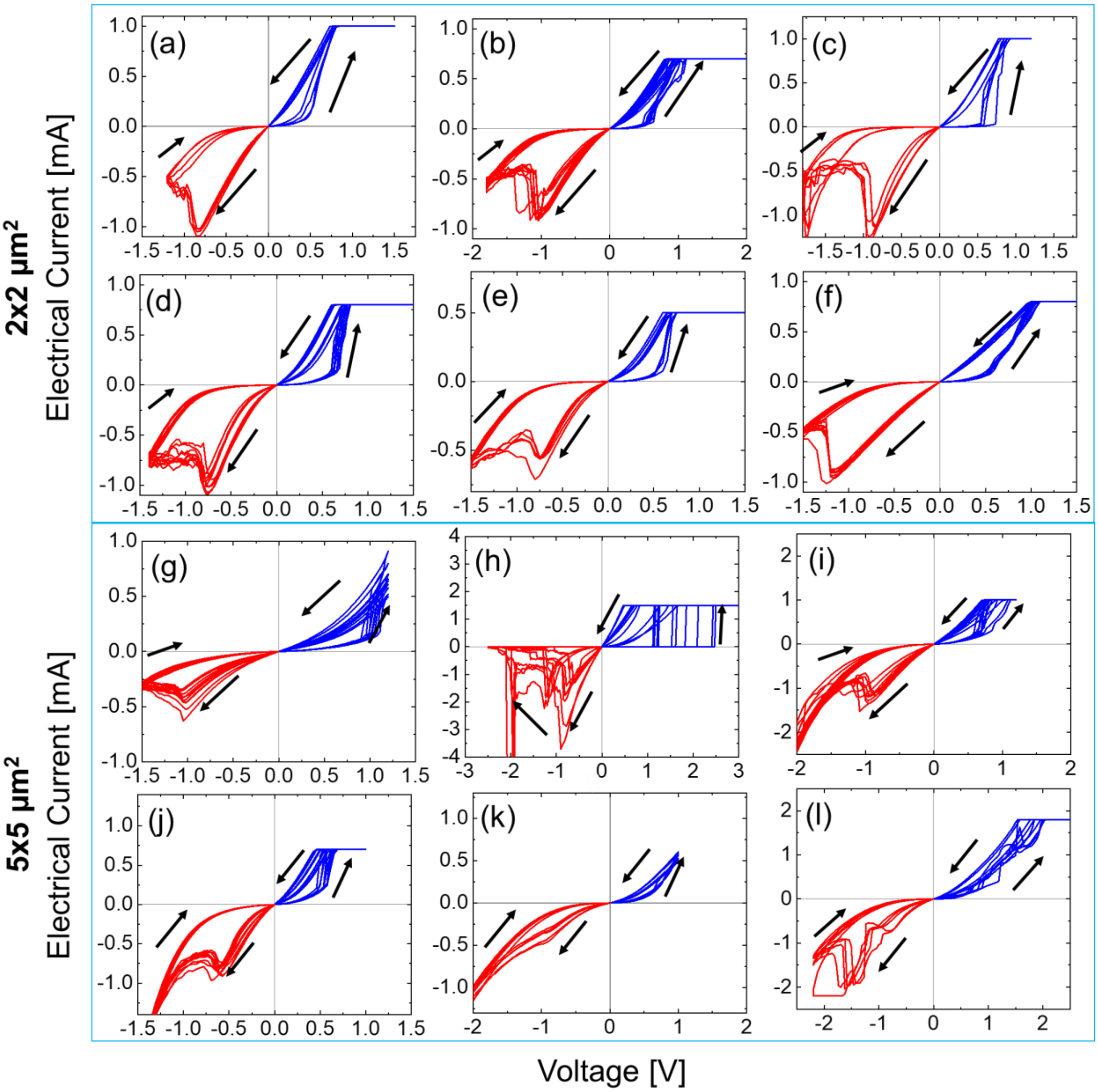

Figure S3. Measured *I-V* cycle characteristics for RRAM devices with a Crosspoint area of **(a-f)** 2x2 µm^2 and **(g-l)** 5x5 µm^2 for six devices each. The blue and the red graphs symbolize the set and reset processes, respectively. The black arrows present the direction of the set and the reset process.

One of the major limitations in RRAM devices is connected with the variability of the *I-V* characteristics. Figure S3 (a-f) show *I-V* cycles in six investigated 2x2 µm^2 devices which we investigated in-operando. In all the six devices we observed a relative stable cycle-to-cycle (C2C) behavior, i.e., the curves of the various cycles matched within the same device. In all the devices we can see a clear trend going from a small slope in the *I-V* curves to a sharp increase at a higher voltage magnitude during the set process. However, these graphs distinguish through the steepness of their slope.

Figure S3 (g-l) show *I-V* cycles in six investigated 5x5 µm^2 devices which we investigated in-operando. On contrary to the 2x2 µm^2 we saw an increased C2C and D2D variability illustrated due to a decay of the current (g), variation of the set voltage and strong reset (h), change of the set slope (i) or by multiple set steps (l). On average we observed a standard deviation of the forming voltage

of 10 % for the 2x2 μm^2 and 19 % for the 5x5 μm^2 devices showing an increased C2C variability in the 5x5 μm^2 devices.

In conclusion, we saw a significant difference in the stability of the *I-V* cycle behavior when comparing the 2x2 and the 5x5 μm^2 devices. The distinct types of set curves of the 5x5 μm^2 devices indicate a non-uniform formation step of the conductive filaments. These results demonstrate the need of thermal characterization of our devices in order to identify the source of the variability in these devices.

S4 Calibration of scanning thermal microscope (SThM)

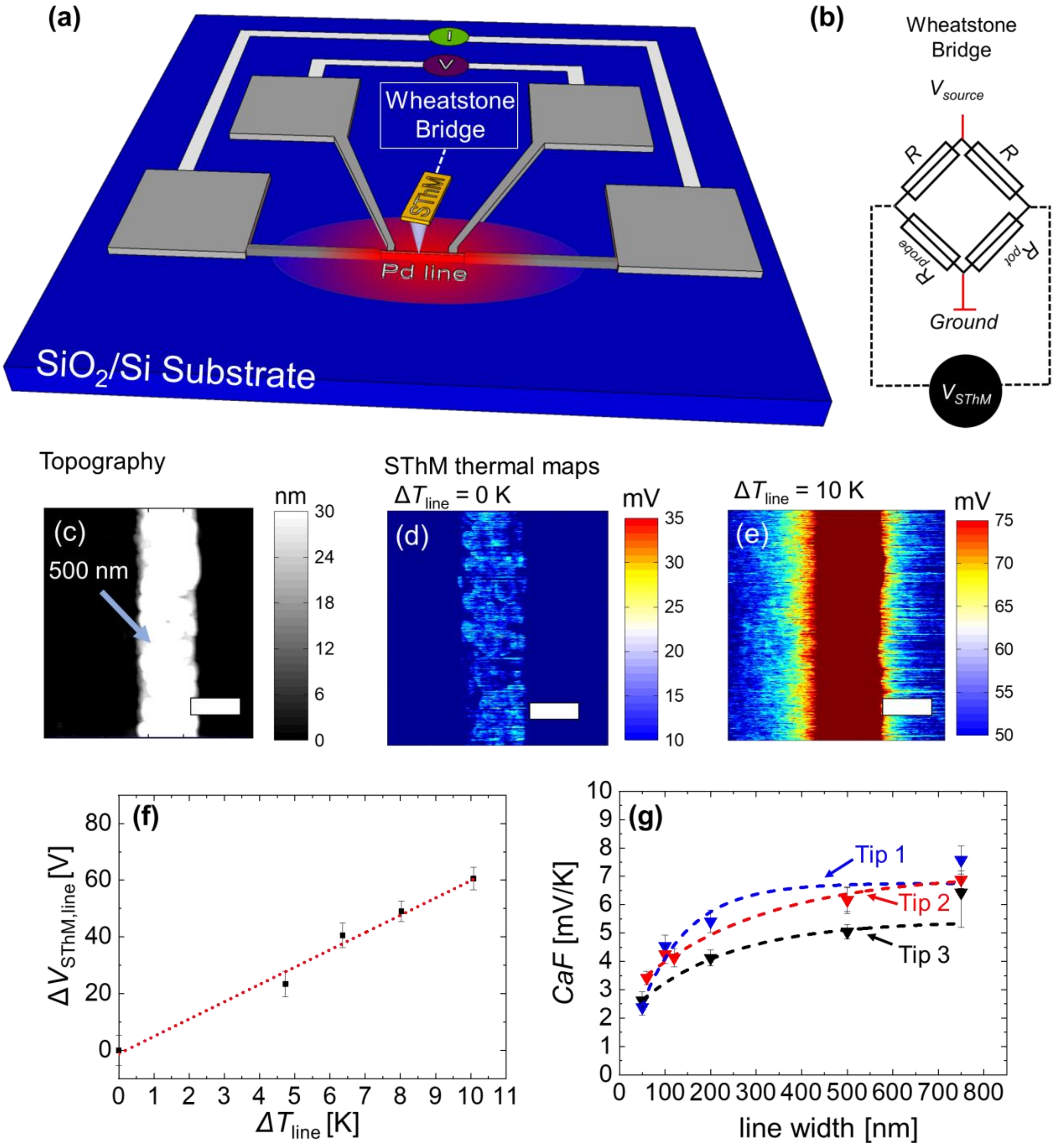

Figure S4. (a) Calibration sample and measurement configurations. Scanning thermal microscope (SThM) probe scans over heated palladium (Pd) lines of different widths (50-750 nm) connected to four Pd heater pads each. The thermoresistive metal lines are characterized electrically by four-Point probe measurements to determine the heating temperature of the lines. **(b)** Design of the Wheatstone bridge connected to the SThM probe during the scan. A constant bridge voltage V_{source} is applied during the measurement and the change in temperature is read due to changes in the bridge signal V_{SThM}. **(c)** Topography image of a 500 nm wide Pd line. **(d,e)** SThM thermal maps of the same line obtained with a temperature increase of the line ΔT_{line} of (d) 0 K (non-heated) and (e) 10 K (heated). (Scale bar is equal to 500 nm). **(f)** SThM thermal Signal of a 500 nm wide Pd line $\Delta V_{SThM,line}$ as a function of ΔT_{line}. **(g)** SThM Calibration Factor (*CaF*) estimated by means of the slope of the $\Delta V_{SThM,line}$ vs ΔT_{line} graphs as a function of the line width for the three different tips we used for the thermal characterization of our devices.

For the calibration of our scanning thermal microscope (SThM) probes, we used an approach as previously applied in literature.[2,3] Figure S4 (a) illustrates the calibration approach which we followed for the SThM thermo-resistive probes used in this study. For the calibration sample, we deposited four 2nm Ti/50 nm Pd heater pads by means of optical lithography and e-beam evaporation. Subsequently we fabricated 2 nm Ti/30 nm Pd metal lines of different widths (50-750 nm) through e-beam lithography and evaporation. To electrically isolate the sample from the probe we further deposited a thin 10 nm Al_2O_3 film on top of the calibration sample using pulsed laser deposition (PLD).[3]

We started with electrically characterizing the different Pd metal lines by applying four-point probe measurements. Therefore, we applied an electric current through the outer heater pads while measuring the voltage drop along the Pd lines through the inner pads, as shown in Figure S4 (a) by means of a semiconductor parameter analyzer (SPA). Pd is a thermo-resistive material meaning its electrical resistance R_{line} is temperature dependent and its value at a temperature T described as follows:

$$R_{\text{line}}(T) = R_{\text{line},0} \cdot (1 + TCR \cdot (T - T_0)) \tag{S1}$$

By means of equation S1 we can estimate the temperature at R_{line} of the line by knowing its temperature coefficient of resistance (TCR) and a reference value $R_{\text{line},0}$ at the temperature T_0. The TCR is a material specific characteristic which we determined by measuring the resistance vs power curves of the lines for different temperatures (room temperature to 340 K) using a temperature adjustable sample stage. Using the TCR we calculated the temperature increase of the line as a function of the power applied to it. A more detailed description of the electrical characterization process can be found in our previous paper.[3]

In the second step we scanned the non-heated and heated metal lines with our SThM probes. In operation the SThM probes were connected to a Wheatstone bridge as illustrated in Figure S4 (b). This electrical network consists of two fixed resistances R (1 kΩ), one adjustable resistance R_{pot} and the resistance of the probe R_{probe}. In operation we applied a voltage across the bridge V_{source} of 0.5 V resulting in a power of 19 µW. In contact with the sample, we nullified the signal measured along the line V_{SThM}. Our SThM probes consists of a thin Pd resistor on top a SiN film. Thus, the resistance of the thermoresistive Pd probe correlates with the temperature of the probe. By that we measured differences in the surface temperature ΔT due to changes in V_{SThM} during the scan.

During the fabrication step we used the nullified SThM probe to estimate the temperature rise of the heated metal lines. As an example, Figure S4 (c) shows the topography map of a 500 nm wide line. In Figure S4 (d) and (e) we plotted two thermal maps obtained on the same line in a (d) non-heated (ΔT_{line} = 0 K) and (e) heated case ($\Delta T_{\text{line}} \approx$ 10 K). In this case we still observed some minor signal variation in the non-heated case at the line, which are originated by differences in the tip to surface interaction. Therefore, we estimated the signal difference at the heated line $\Delta V_{\text{SThM,line}}$ (ΔT_{line}) in comparison with $\Delta V_{\text{SThM,line}}$ (0) at a reference non-heated map to minimize the impact of topographical artefacts. Additionally, we determined the mean value of $\Delta V_{\text{SThM,line}}$ along the line in order to reduce variation of the scan.

By following this principle, we estimated $\Delta V_{\text{SThM,line}}$, measured with the SThM as a function of ΔT_{line} characterized electrically. Figure S4 (f) shows the $\Delta V_{\text{SThM,line}}$ vs ΔT_{line} graph for the 500 nm wide Pd line obtained for five different heating configurations. As in previous studies, we observed a linear correlation of the SThM signal at the line and its temperature.[2,3] Hence, we calculated the calibration factor (*CaF*) of our SThM probes as a function of the line width by determining the slope of each $\Delta V_{\text{SThM,line}}$ vs ΔT_{line} graph.

Figure S4 (g) shows the *CaF* vs line width graphs for the three tips used for the measurements of this study. All of the three graphs show the characteristic saturation behavior of *CaF* for higher line widths as observed before.[2,3] When comparing the three graphs we observed different saturation

values as also differences in the cut off line width of the graphs. Therefore, it is essential to calibrate each probe in order to maximize the accuracy of the calibration process. Each of the three probes are from different patches and therefore can vary in their characteristics.

For example, we obtained that the calibration factor for tip 2 saturated at a value of *CaF* = 7.1 $\pm$ 0.5 mV/K for surface temperature features above 680 nm. This *CaF* was used to calculate the temperatures of most of the hot spots observed experimentally, e.g., for the results in Figure 1 of the main manuscript where the full widths at half maximum (FWHM) of the hot spots exceed the cut-off line width. The electrical signal in mV measured with SThM (ΔV_{SThM}) was calculated as the difference between the heated maps and a reference map at zero power. Overall, ΔV_{SThM} can be converted to the temperature increase (ΔT) in K as follows:

$$\Delta T = \frac{\Delta V_{\mathrm{SThM}}}{CaF} \tag{S2}$$

At this point it is worth noting that a careful calibration allows this approach to be extended for the study of even smaller devices. As can be seen from the graphs in Figure S4 (g) we observe a drop in the calibration factor at a certain cut-off line width. This is related to the disc-shaped thermal exchange area between the tip and the sample. When the size of the device heating feature lies below the size of the cut-off line width, the heat exchange between the tip and device is truncated and the calibration factor decreases. However, beyond the cut-off, one should notice that the conversion factor drastically reduces. For devices or hot spots below ~50 nm determining an accurate value could be very challenging and might also involve considerable uncertainty. Therefore, additional strategies to alleviate it could be to measure in vacuum, i.e. to reduce the thermal exchange radius between the tip apex and the surface by removing heat transfer mechanisms like convection or water meniscus,[4] or to improve the signal processing techniques, like the deconvolution approach described by Deshmukh *et al*.[2]

S5. Steady state SThM characterization

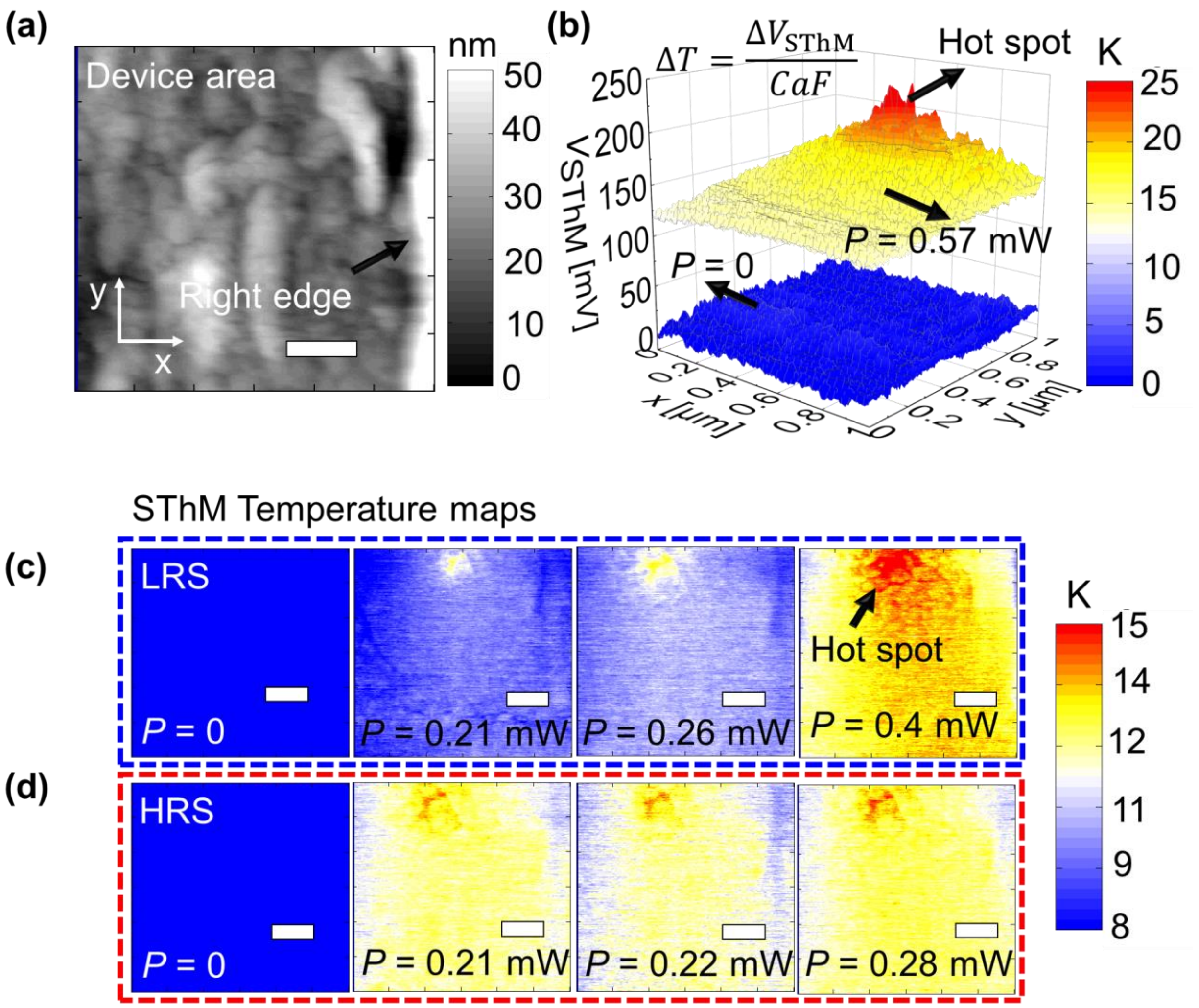

Figure S5. (a) Topographic image of a device with an area of 2x2 µm^2 (scale bar 200 nm) **(b)** 3D SThM temperature V_{SThM} (z-axis) map obtained for the device shown in (a) when applying a power of P = 0.57 mW and under no power applied (P = 0). The difference in SThM signal between heated *vs* non-heated case was converted into a temperature change by using a calibration factor of CaF = 7.1 mV/K. **(c)** and **(d)** SThM temperature maps for four different power magnitudes after (c) setting the device at a positive polarity (blue dashed rectangle) and after (d) resetting the device at the reverse negative polarity (red dashed rectangle). (scale bar 200 nm).

We repeated the procedure described in the main text for the thermal characterization of the hot spot for all our RRAM devices. Figure S5 (a) shows the topography map of a RRAM device with a cross-point area of 2x2 µm^2 obtained with a SThM probe. At this point it is worth mentioning that we observed a variation in the topography roughness of our devices as can be seen when comparing the topography in **Figure S5 (a)** with the one in **Figure 2 (a).** However, we want to clarify that the position of the hot spots is localized independent from any wrinkles or surface roughness variations. Therefore, we estimate that the formation of the filament is not triggered by topographical artifacts. Figure S5 (b) shows a 3D representation of two thermal maps obtained for the same 2x2 µm^2 device. Here we present two maps obtained for a non-heated case (P = 0 mW) and for a heated case (P = 0.57 mW). In the heated case we observed a hot spot induced by current passing through the conductive filament.

Figure S5 (c) shows multiple temperature maps obtained on the same device after the device is set (blue dashed rectangular) vs reset (red dashed rectangular). In the set state we can see that the hot spot is localized at the same location and its temperature scales up as the power applied to the device increases (same set state, i.e., no cycling between images). We observed that the maximum hot spot temperature increased from 13 K at 0.21 mW to 18 K at 0.4 mW. After resetting the device, we carried out temperature maps for similar powers as in the set case but with the reversed polarity (see Figure S5 (d)).

In comparison with the results in Figure 2 of the main text the differences in between the set and reset state are relatively low. By looking at the thermal maps during the reset state we still observed an elevated heating at the initial hot spot position but less localized than in the set state. So, as also observed with a weaker effect in the 5x5 μm^2 device, the filament breakdown is not fully complete. When comparing the set *I*-*V* curves of the two devices in Figure 1 (b) we can see that the difference before and after the set is comparable low in the 2x2 μm^2 device. Therefore, the smaller difference in between set and reset heating might originate from a less complete breakdown.

S6. COMSOL model for the characterization of the filament temperature

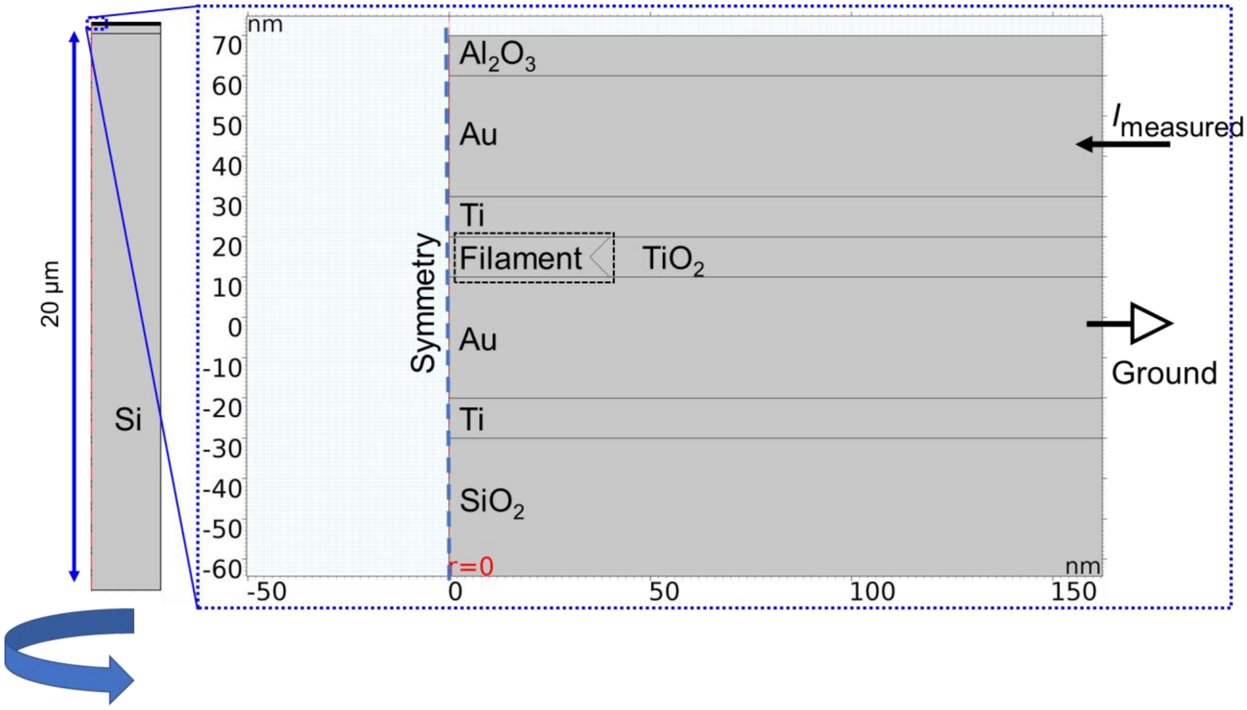

Figure S6. Finite element method (FEM) model geometry of the applied model for the characterization of the hot spot temperature. The model has a cylindrical symmetry around the central axis with a structure equivalent to the experimental TiO_2 RRAM devices with an hourglass shaped filament. An electric current $I_{measured}$ is applied from the top electrode to the bottom electrode.

In order to confirm the experimentally obtained temperatures and to estimate the filament temperature we employed an electrothermal finite element method (FEM) model in COMSOL Multiphysics. We built our model on base considering the same boundary and heat flux conditions of the results obtained in a study by Deshmukh *et al.*[2] By means of that we replicated the structure of the 5x5 µm^2 device which results are shown in Figure 2 of the main text. Therefore, we used a 2.5 x 2.5 x 20 µm model with a cylindrical symmetry around the central axis as shown in Figure S6. We adjusted the geometry to mimic our current Si/SiO_2/Ti/Au/TiO_2/Ti/Au/Al_2O_3 as described in Section S1. As can be seen in Figure S6 we shaped the Filament on the left edge of the TiO_2 using an hourglass structure, as this approach showed the best fitting.

In accordance with previous studies we estimated the thermal contact conductance in between the oxide and the top electrode $G_{TiO2\text{-}Ti/Au}$ using the full width at half maximum (FWHM) of our SThM scans of the hot spot as follows.[2] The thermal healing length of the hot spot is

$$L_{\mathrm{H}} = \sqrt{\frac{k_{\mathrm{th,TE}} \cdot t_{\mathrm{TE}}}{G_{\mathrm{TiO2-Ti/Au}}}} \quad \text{(S3)}$$

where $k_{th,TE}$ and t_{TE} are the thermal conductivity and thickness of the top electrode, respectively. In our RRAM devices we can estimate FWHM ≈ $2L_H$ given the small size of our filaments ($d_{CF} \ll L_H$). Therefore, we estimated the FWHM of the hot spot temperature peaks for the three images investigated. The thickness and thermal conductivity of the top electrode as also the remaining material characteristics are assembled from the literature and the COMSOL library. Table S1 shows the fixed material and contact parameters used for this FEM simulation. It is worth noting that one can reduce the heat spreading from the filament to the surface by reducing the thickness of the top electrode. On the other hand, it is also important to take into account that another key restricting factor for this heat spreading is the thermal interface between the filament and the top electrode, as discussed by Deshmukh et al.[2]

For the characterization of the Joule heating, we adjusted the top electrode as a current source with an applied current equal to the values measured during the SThM mapping $I_{measured}$. The bottom electrode acts as the electrical ground at which the potential is 0 V.

Table S1. Model Parameters used in the COMSOL Simulation

Parameter	Value at room temperature	Reference
σ_{Au}	**14.28·10^{6} S/m**	[7]
k_{Au}	**90 W/(m·K)**	[7]
k_{TiO2}	**0.8 W/(m·K)**	[8]
k_{Ti}	**8.2 W/(m·K)**	[9]
$G_{TiO2-Ti/Au}$	**1.25 MW/(m^{2}·K)**	[2]
σ_{TiO2}	**10^{-15} S/m**	[2]
k_{SiO2}	**1.4 W/(m·K)**	COMSOL Database
k_{Si}	**150 W/(m·K)**	[2]
k_{Al2O3}	**3 W/(m·K)**	[2]
$G_{SiO2-Ti/Au}$	**80 MW/(m^{2}·K)**	[10]
$G_{SiO2-Si}$	**434 MW/(m^{2}·K)**	[2]

S7. Estimation of the hot spot characteristics

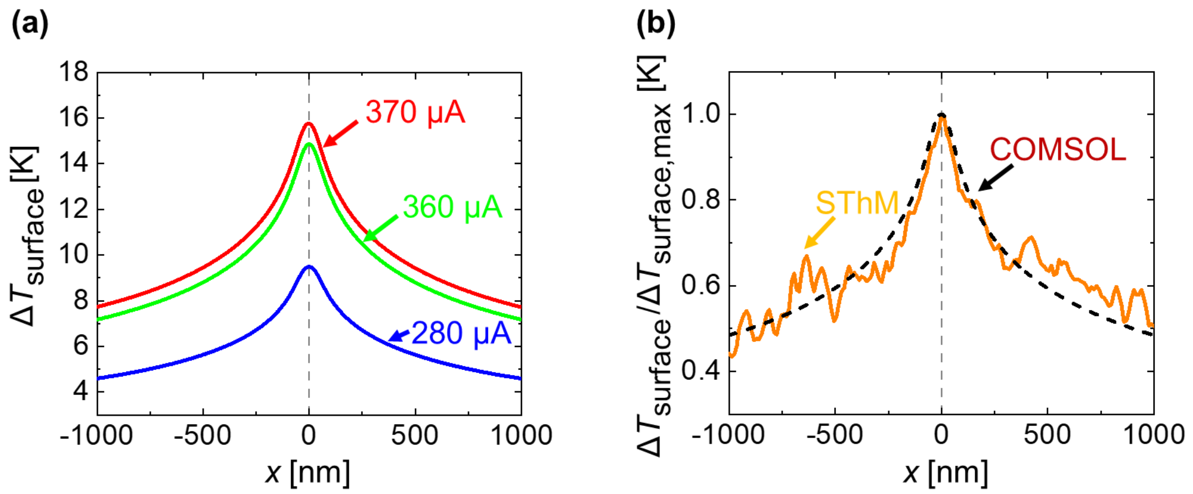

Figure S7. (a) Simulated temperature increase along the surface $\Delta T_{surface}$ of the 5x5 µm^2 RRAM device structure for three different currents $I_{measured}$ (280 µA in blue, 360 µA in green and 370 µA in red). **(b)** Normalized simulated (dashed line) and measured SThM (straight line) temperature increase $\Delta T_{surface}/\Delta T_{surface,max}$ along the surface of the RRAM device for an electric current $I_{measured}$ of 280 µA.

For the estimation of the hot spot characteristics, we adjusted the remaining model parameters in order to fit the surface temperature, FWHM and the electrical potential measured during the SThM scans. The sweep parameter included the size characteristics of the filament (top radius, bottom radius and the center radius) the thermal conductivity of the filament $k_{th,filament}$ the thermal contact conductance in between the filament and the top and bottom electrode $G_{CF\text{-}TE}$ and $G_{CF\text{-}BE}$ respectively and the electrical contact resistance in between the filament and the top electrode $\rho_{c,\ CF\text{-}TE}$. The initial values for these parameters were estimated on base of the simulation characteristics displayed in ref.[2]

Table S2. Comparison of the hot spot characteristics of the SThM measurements and the COMSOL Multiphysics simulation for the three investigated scans. Also shown are the corresponding sweep parameters that showed the best fits.

**Characteristic**	**$I_{measured}$ = 280 µA**	**$I_{measured}$ = 360 µA**	**$I_{measured}$ = 370 µA**
**Experimental observations**			
**FWHM [nm]**	**1820**	**1920**	**1820**
**$\Delta T_{max,surface}$ [K]**	**8.62**	**12.52**	**13.9**
**Potential [V]**	**0.41**	**0.49**	**0.54**
**COMSOL Multiphysics results**			
**FWHM [nm]**	**1880**	**1820**	**1820**
**$\Delta T_{max,surface}$ [K]**	**9.48**	**14.75**	**15.82**
**Potential [V]**	**0.41**	**0.49**	**0.54**
**$\Delta T_{max,filament}$ [K]**	**171.61**	**238.85**	**245**
**Sweep Parameter**			
**$k_{th,\ filament}$ [W/(m·K)]**	**3**	**3**	**3**
***Bottom radius* [nm]**	**43**	**44**	**47**
***Top radius* [nm]**	**41**	**43**	**43**
***Center radius* [nm]**	**38.5**	**41**	**41.5**
**$G_{CF\text{-}TE}$ [MW/(m^2·K)]**	**12**	**12**	**12**
**$G_{CF\text{-}BE}$ [MW/(m^2·K)]**	**100**	**100**	**100**
**$\rho_{C,\ CF\text{-}TE}$ [Ω·cm^2]**	**4.76 · 10^{-8}**	**5 · 10^{-8}**	**5.26 · 10^{-8}**

Table S2 shows the sweep parameter and the results of the COMSOL simulation which presented the best fit with the experimental results. Figure S7 (a) shows the corresponding simulated temperature profiles at the surface of the RRAM devices for the best fitting parameters of the three investigated SThM maps. In general, we observed a good fit of the FWHM and the potential drop in between experiments and simulation. We obtained slightly higher (10-15 %) values for the maximum temperature at the surface $\Delta T_{max,surface}$ from the simulation in comparison with the experimental results. Considering the error of our calibration factor and the other material parameters this temperature difference is in a reasonable range. Figure 7 (b) shows a fit of the experimentally obtained surface temperature profile (straight line) with the profile obtained from the COMSOL simulation (dashed line) for $I_{measured}$ = 280 µA. In this graph we normalized the temperature in order to take the temperature difference into account.

It is worth noting that heat dissipation depends on the device area. For wider area devices, electrodes can carry away heat more easily which leads to a decrease in the maximum surface temperature under identical thermal interfaces, filament size and electrode thermal conductivity.

In comparison to the results of Deshmukh *et al.*[2] we estimated a higher $k_{th,filament}$, which can be originated from the difference in the material characteristics and higher thickness of the titanium oxide layer and thus the filament. Additionally, our simulations results indicate a relatively high filament diameter of around 41-44 nm, which is connected to the relatively high electric currents applied to the device. Finally, the maximum temperature increases of the filaments $\Delta T_{max,filament}$ is estimated to be in between 172 and 245 K, correlating with the power applied to the device.

S8. Hot spot location with cycling

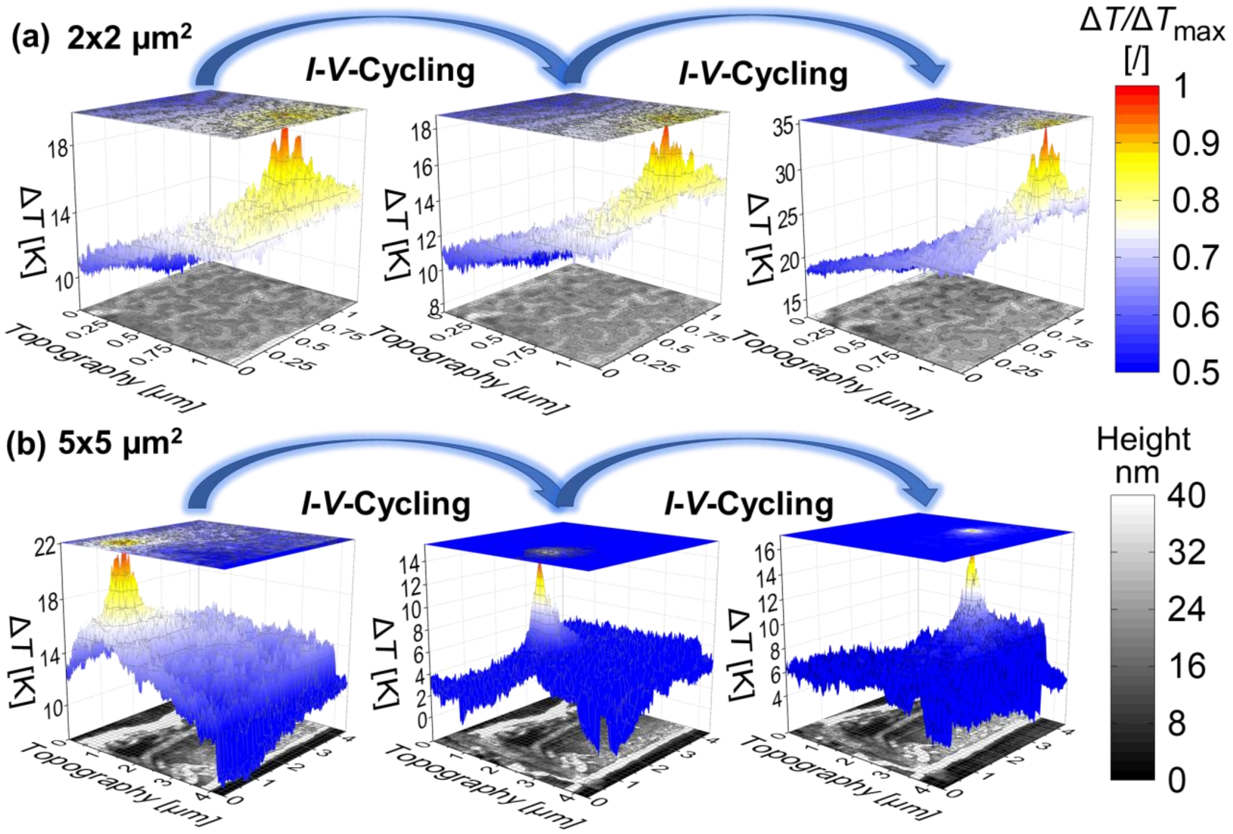

Figure S8. 3D surface temperature maps (z-axis) plotted over the 2D topography (x and y axis) of devices with an area of **(a)** 2x2 µm^2 and **(b)** 5x5 µm^2. The 2D topographic maps at the bottom show the height differences in the scanned area (greyscale bar on the bottom right). The color scheme of the 3D plots shows the temperature increase during the steady state measurements (colored bar on the top right). Power *P* applied to the devices during the scans in (a) are 0.33 mW/0.32 mW/0.59 mW and in (b) are 0.27 mW/0.55 mW/0.47 mW from left to right.

For the characterization of the hot spot moveability, we repeated the steady state measurements of the devices after *I*-*V* cycling, Figure S8 (a) and (b) show two examples of heat dissipation in set devices at different stages of cyclability for a (a) 2x2 µm^2 and (b) 5x5 µm^2 device. In addition to the maps of Figure 4 of the main text, we observed a static hot spot position in the 2x2 µm^2 device while a shift of the hot spot occurred in the 5x5 µm^2 device.

S9. Characterization of the line resistance

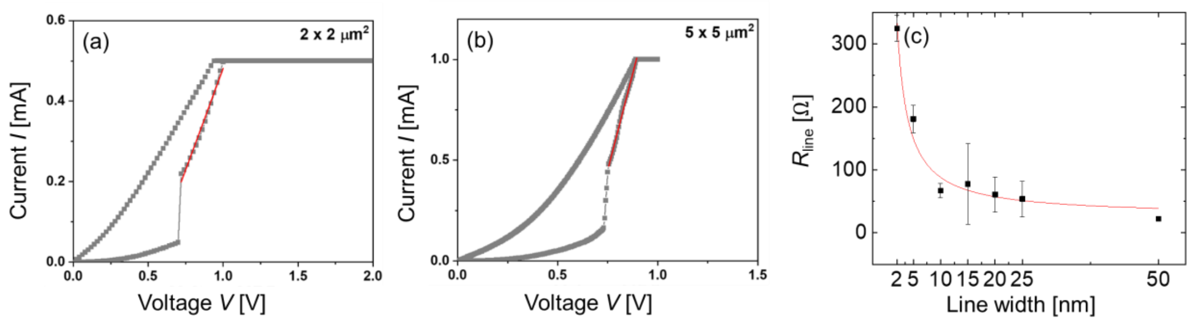

Figure S9. (a) and (b) Measured *I-V* characteristics of the 2x2 μm^2 and 5x5 μm^2 devices, respectively. The red line is the fit of the linear region. (c) Measured line resistance R_{line} of the bottom and top electrode as a function of the line width of the device.

The *I-V* curves we measured are the extrinsic *I-V* characteristics. The voltage drops not only on the memory cell but also on the series resistance, which includes the line resistance of the electrodes, the contact resistance of the probes and pads and other contributions from the device stack, as for example the vertical conduction in the electrodes. By subtracting the voltage drop over the series resistance, the intrinsic *I-V* characteristics can be obtained.[5] Fantini et al.[5] reported that the intrinsic *I-V* behavior of resistive switching devices shows that the set is triggered at a certain threshold voltage and is followed by a snapback to a voltage value where the differential resistance *dV/dI* is approximately 0, i.e. a vertical line in the *I-V* characteristics. Taking this into account, the series resistance can be estimated by calculating the series resistance value that transforms the extrinsic *I-V* curve into the intrinsic one, exhibiting the vertical transition behavior.

We extracted the approximate value of the series resistance for 2x2 μm^2 and 5x5 μm^2 devices from the extrinsic *I-V* curves in Figure S9 based on the abovementioned method. During the set process, after the abrupt jump at the threshold voltage, the current shows a linear dependence with the voltage before reaching the compliance. This is caused by the series resistance. Its value can be estimated from the slope of the curve in the linear region (the red line in Figure S9). The estimated series resistance of 2x2 μm^2 and 5x5 μm^2 devices is 1 kΩ and 300 Ω, respectively. The contact resistance between the probe and the electrode is similar due to the same measurement method, therefore the line resistance and the other electrode contributions should be the dominating cause of the difference in the series resistance. To be noted, these values are not for the pristine devices, but for the devices that switched for at least 20 cycles.

Additionally, we measured the line resistance of both the top and bottom electrode for various pristine devices. Figure S9 (c) shows the measured combined line resistance R_{line} of the bottom and top electrode as a function of the line width. At a higher line width, we observed a relatively constant value for R_{line}. In these cases, we expect the contact resistance between the measuring probes and the electrode surface to be the dominating factor of R_{line}. For the relevant line widths (2 μm and 5 μm) we measured an increment in the line resistance based on the decreased line area. By subtracting the estimate of the contact resistance, we obtained a R_{line} of 123 Ω and 308 Ω for the 5x5 and 2x2 μm^2 devices, respectively. The difference from the measured line resistance and the estimated series resistance stems from the contact resistance between the lines and the active area and possible other resistances which are not included in the line resistance.

S10. STEM characterization of the layer structure in pristine and cycled devices

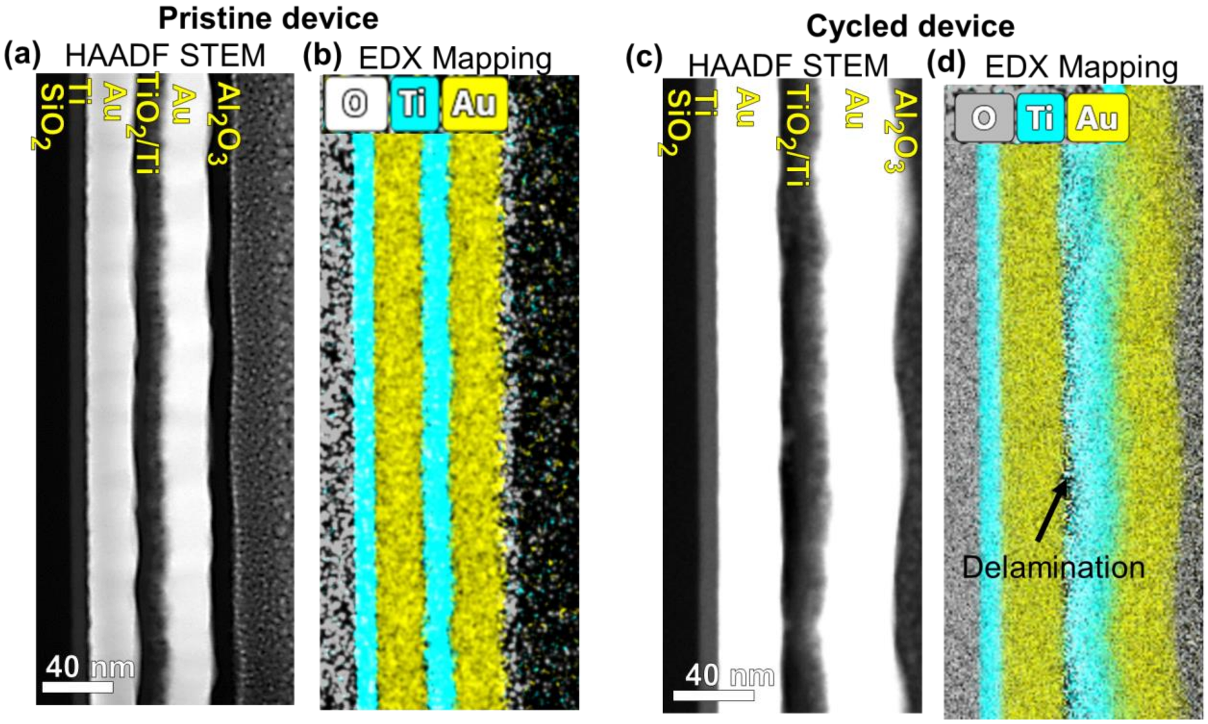

Figure S10. (a) High-angle annular dark field scanning transmission electron microscopy (HAADF STEM) images of a crosspoint device area of one pristine 5x5 µm^2 TiO_2 RRAM device. (b) Energy-dispersive X-ray spectroscopy (EDX) images showing the gold (gold), titanium (light blue) and oxygen (grey) compositions of the same area as in (a). (c) High-angle annular dark field scanning transmission electron microscopy (HAADF STEM) images of a cross-point device area of one cycled 5x5 µm^2 TiO_2 RRAM device. (d) Energy-dispersive X-ray spectroscopy (EDX) image showing the Gold (gold), Titanium (light blue) and oxygen (grey) compositions of the same area as in (c).

In order to investigate the impact of the electrical forming and cycling of the device on the material structure we employed high-angle annular dark field scanning transmission microscopy (HAADF-STEM) with an energy-dispersive X-ray spectroscopy (EDX) detector. First, we cut the samples out of the cross-point area of our RRAM devices using the focused ion beam (FIB) technique. Second, we employed transmission electron microscopy (TEM) in the cross-section direction for the characterization and evaluation of the device layer structure. By means of that we aimed to evaluate the chemical composition and distribution of the three main elements i.e., Au, Ti and O which are the building blocks for the metal/insulator/metal (Au/TiO_2/Ti/Au) structure of our RRAM devices.

Figure S10 (a) and (b) show the HAADF and the EDX images obtained from a pristine, i.e., not electrically formed device. Hereby, the HAADF imaging method enables us to clearly identify the high atomic number Au electrodes. The remaining layers are indicated in Figure S10 (a) according to the device structure. We clearly observed a continuous Ti and O signal along the TiO_2/Ti areas in between the electrodes.

Figure S10 (c) and (d) show the HAADF and the EDX images obtained of an already cycled 5x5 µm^2 device. Similar to the pristine device the EDX image shows a clear Au signal at the position of the electrodes. However, we observed a small but not continuous drop of the Ti and O signal at the transition area in between the TiO_2 layer and the bottom electrode.

Similar observations have been made by Carta *et al.*[6] in a Pt/TiO_2/Pt RRAM device. In their study, they investigated the material structure of their RRAM devices in pristine and in formed state. They performed HAADF-STEM measurements with an EDX detector for the characterization of the

devices before and after electrical cycling. On the one hand they observed a continuous Ti and O signal in the pristine device. On the other hand, the measurements revealed a drop in the Ti and O signal in between the TiO_2 layer and the Pt top electrode in the formed devices referred to delamination. This delamination effect was claimed to be originated from the development of O_2 gas during the formation of the conductive filament.

From our results we cannot make a solid conclusion about the impact of the layer delamination observed in our devices on their *I*-*V* or heating variation during cycling. However, we should emphasize that this effect might lead to a limitation in the electrical performance and variability of RRAM devices. Presumably, the delamination should lead to a reduction of the effective cross-point area of the devices.

S11. *I-Vs* and thermal data for multiple cycles

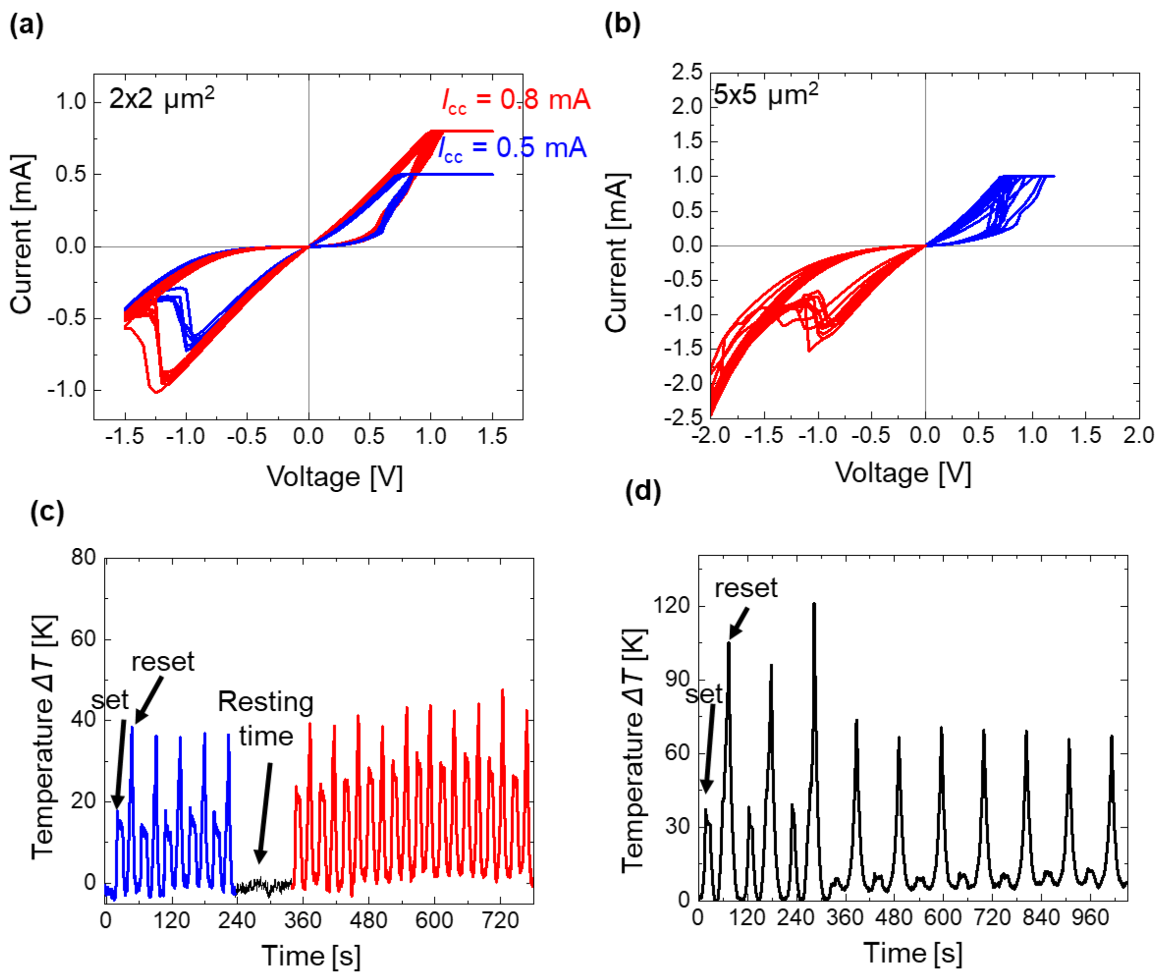

Figure S11. (a) and (b) *I-V* curves for 15 and 10 cycles for 2x2 µm^2 and a 5x5 µm^2 devices, respectively. (c) and (d) logger temperature measured for the different set and reset cycles of the devices shown in (a) and (b), respectively, at same SThM probe location.

Figure S11 shows two examples of *I-V* graphs and the corresponding SThM-temperature logger graphs of a 2x2 µm^2 and a 5x5 µm^2 device. Figure S11 (a) and (b) show the current vs voltage behavior of the devices during cycling. Figure S11 (c) and (d) show the temperature evolution at the initial hot spot location measured simultaneously during the *I-V* cycling for 15 and 10 full cycles, respectively. The cycling behavior of the 2x2 µm^2 device remains constant, accompanied by a consistent series of set-reset temperature evolutions in the logger graphs, indicating a static hot spot location. This can also be observed at different current compliances I_{cc} (blue I_{cc} = 0.5 mA vs red I_{cc} = 0.8 mA). However, in the 5x5 µm^2 device we observe a drastic change of the temperature evolution in accordance with the variability of the *I-V* curves. The device still remains cyclable, but the temperature becomes lower after a certain number of cycles. These observations indicate that a new filament is eventually formed at a different location. This behavior can be extrapolated to more cycles, simply based on the reliability of the device. As we show in supporting information section S2, our devices remain cyclable after 50 switching cycles.

Supporting information references

(1) Wong, H. P.; Lee, H.; Yu, S.; Chen, Y.; Wu, Y.; Chen, P.; Lee, B.; Chen, F. T.; Tsai, M. Metal – Oxide RRAM. *Proc. IEEE* **2012**, *100*, 1951–1970.

(2) Deshmukh, S.; Muñoz Rojo, M.; Yalon, E.; Vaziri, S.; Köroğlu, Ç.; Islam, R.; Iglesias, R. A.; Saraswat, K.; Pop, E. Direct Measurement of Nanoscale Filamentary Hot Spots in Resistive Memory Devices. *Sci. Adv.* **2022**, *8*, eabk1514.

(3) Swoboda, T.; Wainstein, N.; Deshmukh, S.; Köroğlu, Ç.; Gao, X.; Lanza, M.; Hilgenkamp, H.; Pop, E.; Yalon, E.; Muñoz Rojo, M. Nanoscale Temperature Sensing of Electronic Devices with Calibrated Scanning Thermal Microscopy. *Nanoscale* **2023**, *15* (15), 7139–7146.

(4) Zhang, Y.; Zhu, W.; Hui, F.; Lanza, M.; Borca-Tasciuc, T.; Muñoz Rojo, M. A Review on Principles and Applications of Scanning Thermal Microscopy (SThM). *Adv. Funct. Mater.* **2020**, *30* (18), 1900892.

(5) Fantini, A.; Wouters, D. J.; Degraeve, R.; Goux, L.; Pantisano, L.; Kar, G.; Chen, Y. Y.; Govoreanu, B.; Kittl, J. A.; Altimime, L.; Jurczak, M. Intrinsic Switching Behavior in HfO 2 RRAM by Fast Electrical Measurements on Novel 2R Test Structures. In *2012 4th IEEE International Memory Workshop*; IEEE, 2012; pp 1–4.

(6) Carta, D.; Salaoru, I.; Khiat, A.; Regoutz, A.; Mitterbauer, C.; Harrison, N. M.; Prodromakis, T. Investigation of the Switching Mechanism in TiO2-Based RRAM: A Two-Dimensional EDX Approach. *ACS Appl. Mater. Interfaces* **2016**, *8* (30), 19605–19611.

(7) Mason, S. J.; Wesenberg, D. J.; Hojem, A.; Manno, M.; Leighton, C.; Zink, B. L. Violation of the Wiedemann-Franz Law through Reduction of Thermal Conductivity in Gold Thin Films. *Phys. Rev. Mater.* **2020**, *4* (6), 065003.

(8) Scott, E. A.; Gaskins, J. T.; King, S. W.; Hopkins, P. E. Thermal Conductivity and Thermal Boundary Resistance of Atomic Layer Deposited High- k Dielectric Aluminum Oxide, Hafnium Oxide, and Titanium Oxide Thin Films on Silicon. *APL Mater.* **2018**, *6* (5), 058302.

(9) Sandell, S.; Maire, J.; Chávez-ángel, E.; Torres, C. M. S.; Kristiansen, H.; Zhang, Z.; He, J. Enhancement of Thermal Boundary Conductance of Metal–Polymer System. *Nanomaterials* **2020**, *10* (4), 670.

(10) Duda, J. C.; Yang, C. Y. P.; Foley, B. M.; Cheaito, R.; Medlin, D. L.; Jones, R. E.; Hopkins, P. E. Influence of Interfacial Properties on Thermal Transport at Gold:Silicon Contacts. *Appl. Phys. Lett.* **2013**, *102* (8), 081902.
